# Steroids, lidocain and ioxaglic acid modify the viscosity of hyaluronic acid: in vitro study and clinical implications

**DOI:** 10.1186/s40064-016-1762-z

**Published:** 2016-02-24

**Authors:** Thierry Conrozier, Jeremy Patarin, Pierre Mathieu, Marguerite Rinaudo

**Affiliations:** Service de rhumatologie, Hôpital Nord Franche-Comté, 14 rue de Mulhouse, 90000 Belfort, France; Rhéonova, Domaine Universitaire, 363, rue de la chimie, 38400 Saint Martin d’Hères, France; Laboratoire de Rhumatologie Appliquée, 19 place Tolozan, 69001 Lyon, France; Biomaterials Applications, 6 rue Lesdiguières, Grenoble, France

**Keywords:** Hyaluronic acid, Intra-articular corticosteroid, Triamcinolone, Cortivazol, Lidocain, Ioxaglic acid, Contrast product, Viscosupplementation, Rheology

## Abstract

**Background:**

Viscosupplementaion by intra-articular injection of hyaluronic acid (HA) is a therapeutic modality for treating osteoarthritis of the knee, of the hip and less frequently of other joints. During viscosupplementation, it is usual to inject other drugs, without knowing whether this association may have a deleterious effect thereon. The rheological properties of a viscosupplement are highly dependent on the product [molecular weight × concentration] of HA. Therefore, any reduction of its viscoelastic properties is related either to a decrease of its concentration or/and of its molecular weight. The presence of other molecules can create favorable or unfavorable molecular interactions with HA. The objective of the study was to investigate the effect of products, that are commonly associated with HA (corticosteroids, lidocain, iodinated contrast media), on the rheological behavior of HA, then to try drawing practical conclusions.

**Methods:**

The rheological behavior of both a linear and a cross-linked HA, was studied before and after mixing with different volumes (ratio 1:0.5–1:4) of the following compounds: phosphate buffered saline (PBS, as a control), cortivazol, triamcinolone hexacetonide, lidocain chlorhydrate and meglumine ioxaglate. The flow curve of the different samples was obtained using a measuring method based on a constant shear rate.

**Results:**

Whatever the dilution and the added molecule were, viscosity of the cross-linked viscosupplement remained much higher than that of the linear one. Addition of PBS at a ratio 1:1 caused a dramatic decrease (up to 97.5 %) of HA viscosity. Cortivazol and lidocain had a similar effect than PBS on linear HA. Both were much deleterious on cross-linked HA viscosity. Among corticosteroids, triamcinolone decreased much less HA viscosity than cortivazol. The effect of meglumine ioxaglate was dose-dependent. Up to a ratio 1:1 viscosity of the linear HA remained above the dilution effect. On the cross-linked HA, the deleterious effect of the contrast agent was evident as soon as a ratio 1:1 and became very marked at 1:2.

**Conclusion:**

HA viscosity varies widely in presence of other molecules. These changes are due to both dilution and molecular interactions. This study suggests that addition of other molecules with HA can lead to a major decrease of its viscosity. However, provided to respect a maximum ratio of 1:1, the contrast medium and triamcinolone seem to have no major deleterious effect on the viscosity level, especially on crosslinked HA. The study also suggests a deleterious effect of lidocain on the cross-linked HA. These in vitro data suggest that drugs associations must be avoided when they are not essential. However, clinical trials are needed to determine whether these rheological changes may have a significant impact on the clinical outcome.

## Background

Viscosupplementation by intra-articular injection(s) of hyaluronic acid (HA) is a symptomatic treatment, widely used for treating osteoarthritis of the knee (Legré-Boyer 2015; Bruyère et al. 2014; Bannuru et al. 2015) and, to a lesser extent, of the hip, ankle, shoulder, and trapezio-metacarpal joints. Whatever the joint to be treated, the therapeutic protocol is usually the same: 3–5 injections, 1 week apart, if a linear viscosupplement is used (initial structure of the HA molecule) or 1–3 injections when using a cross-linked HA. To date cross-linking is the main means for increasing the intra-articular residence time of the viscosupplement and therefore for reducing the number of injections (Lindenhayn et al. 1997; Lindqvist et al. 2002). The injected HA is assumed to induce synthesis of endogenous hyaluronic acid (Bagga et al. 2006) which properties are primarily anti-degradative and slightly anti-inflammatory. (du Souich 2014; Henrotin et al. 2013; Li et al. 2012). Compared to corticosteroids, HA acts in a more prolonged but also more delayed way (Bannuru et al. 2009). Therefore, it is common that a steroid is injected with the HA, for pain relief the first 4 weeks during which the HA has not yet had time to act. Although a synergistic effect of the two molecules has been suggested (Grecomoro et al. 1992; Ozturk et al. 2006; de Campos et al. 2013) the impact of corticosteroids on the behavior of HA solutions has never been studied. Some practitioners also use a local anesthetic, as an analgesic to alleviate pain due to the injection, or in a goal to ensure the proper intra-articular needle position when the injection is performed using anatomical landmarks or under ultrasound guidance (Qvistgaard et al. 2001). Again there is no data on the impact of local anesthetics on the visco-elastic behavior of HA.

Finally, most of the studies assessing the accuracy of intra-articular injections conclude at very a high failure rate when they are not performed under imaging guidance (Schumacher 2003; Hall 2013; Kurup and Ward 2010) or by highly trained operators (Mei-Dan et al. 2013). For this reason, in all joints, excepted the knee, it is advisable to carry out the HA injections under ultrasound or radiological control to optimize the chances of successful treatment. If the injections are performed under fluoroscopic control, iodinated contrast use is recommended, but again, the available data, on both the effect of the contrast agents on HA and the optimal volume to be injected, are lacking and do not allow to propose recommendations in this area.

The objective of this study was to investigate in vitro the impact of different products, frequently associated with HA (injectable steroids, local anesthetics and iodinated contrast agent) on the rheological properties of HA, then to try drawing practical conclusions.

## Methods

### Studied devices and drugs

Two viscosupplements were assessed. Hanox-M (Happyvisc^®^, LABRHA, Lyon, France) is a linear HA, MW 1.5 MDa, 31 mg/2 ml, combined with mannitol (3.5 %). Hanox-M-XL (Happycross^®^, LABRHA, Lyon, France) is a cross-linked HA, 35.2 mg/2.2 ml, combined with mannitol (3.5 %).

Two intra-articular corticosteroids were studied: cortivazol 3.75 mg/1.5 ml (Altim^®^, Laboratoire Sanofi-Aventis, Paris, France) and triamcinolone hexacetonide 40 mg/2 ml (Hexatrione^®^, laboratoires DEXO, Saint Cloud, France).

The studied anesthetic was lidocain chlorhydrate 10 mg/ml (Laboratoire Aguettant, Saint-Fons, France).

The contrast agent was the oxaglic acid, meglumine salt, 320 mg Iode/ml (Hexabrix^®^ 320 mg, Laboratoire GUERBET, Roissy Charles de Gaulle, France).

### Experimental methods

For each of the 2 studied HA, 4 solutions were prepared by mixing 1 volume of HA with 0.5, 1, 2, and 4 volumes of phosphate buffered saline (PBS), to obtain a control solution and to measure only the effect of dilution on the rheological properties of HA. Solutions were then made by mixing 1 ml of both HA with variable volumes (ranging from 0.5 to 4 ml) of the studied drugs. To get closer as possible to the clinical practice, studied ratios were 1:1 for corticosteroids, 0.5:1, 1:1, 1:2 and 1:4 for the contrast agent and 1:1 and 1:2 for the local anaesthetic.

Before starting measurements, the sample was set on the bottom plane of the rheometer and squeezed using the upper cone at the measurement gap. Product surplus was removed with a spatula or syringe. The sample was conditioned at a temperature of 25 °C with a precision of 0.5 °C. The different samples flow curves were obtained using a measurement protocol based on a several steps of steady flow at constant shear rate. The stress and shear viscosity values were monitored since the flow was considered established at each step.

To compare the direct effect of the solvent on the dilution, the flow curves of each HA were also established with different products for a same dilution. The results were given with an accuracy of 10 % due to the precision of the measurement system.

## Results

### Influence of dilution

**Fig. 1:**
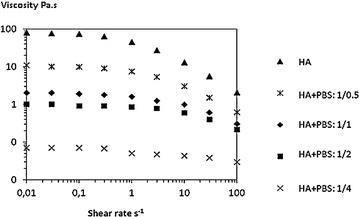
Flow curve of linear hyaluronic acid diluted with increasing volumes of PBS. Viscosity is given in Pa.s according to the shear rate s^−1^. *HA* hyaluronic acid, *PBS* phosphate buffer saline

Diluted by PBS, linear HA preserved its shear-thinning behaviour and exhibited a non-Newtonian plateau. Nevertheless, viscosity levels decreased very significantly as soon as a 1:0.5 ratio, as shown by a 13 % residual viscosity, reaching only 2.5 % at a 1:1 ratio, at gradient speed 0.01 s^−1^ (Fig. 1; Table 1).

**Table 1:** Viscosity (Pa.s) of hyaluronic acid (HA) solutions at 0.01 s^−1^ and 25 °C according to dilution by phosphate buffer (PBS)

HA structure	Viscosity at 0.01 s^−1^ (Pa.s)				
	Initial	Ratio 1/0.5	Ratio 1/1	Ratio 1/2	Ratio 1/4
Linear HA	77	10	2	1	0.07
Cross-linked HA	2560	1010	520	130	15

The rheological behavior of the cross-linked HA remained non-Newtonian in all cases.

**Fig. 2:**
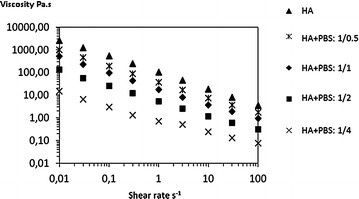
Flow curve of cross-linked hyaluronic acid diluted with increasing volumes of PBS. Viscosity is given in Pa.s according to the shear rate s^−1^. *HA* hyaluronic acid, *PBS* phosphate buffer saline

However viscosity levels decreased with increasing dilution, but in a lower proportion than for the linear product, in particular at ratio 1:0.5 and 1:1, where the viscosity at 0.01 s^−1^ was still 39 and 20 % of the non-diluted viscosity respectively (Fig. 2; Table 1). The cross-linked HA, initially much more viscous than the linear HA, maintained a residual viscosity at a dilution of 1/2 twice as great as the undiluted linear product.

This experience shew clearly that dilution is a very important factor, greatly for the viscosities of linear HA solutions and lesser for cross-linked HA solutions.

### Influence of addition of different diluents

**Fig. 3:**
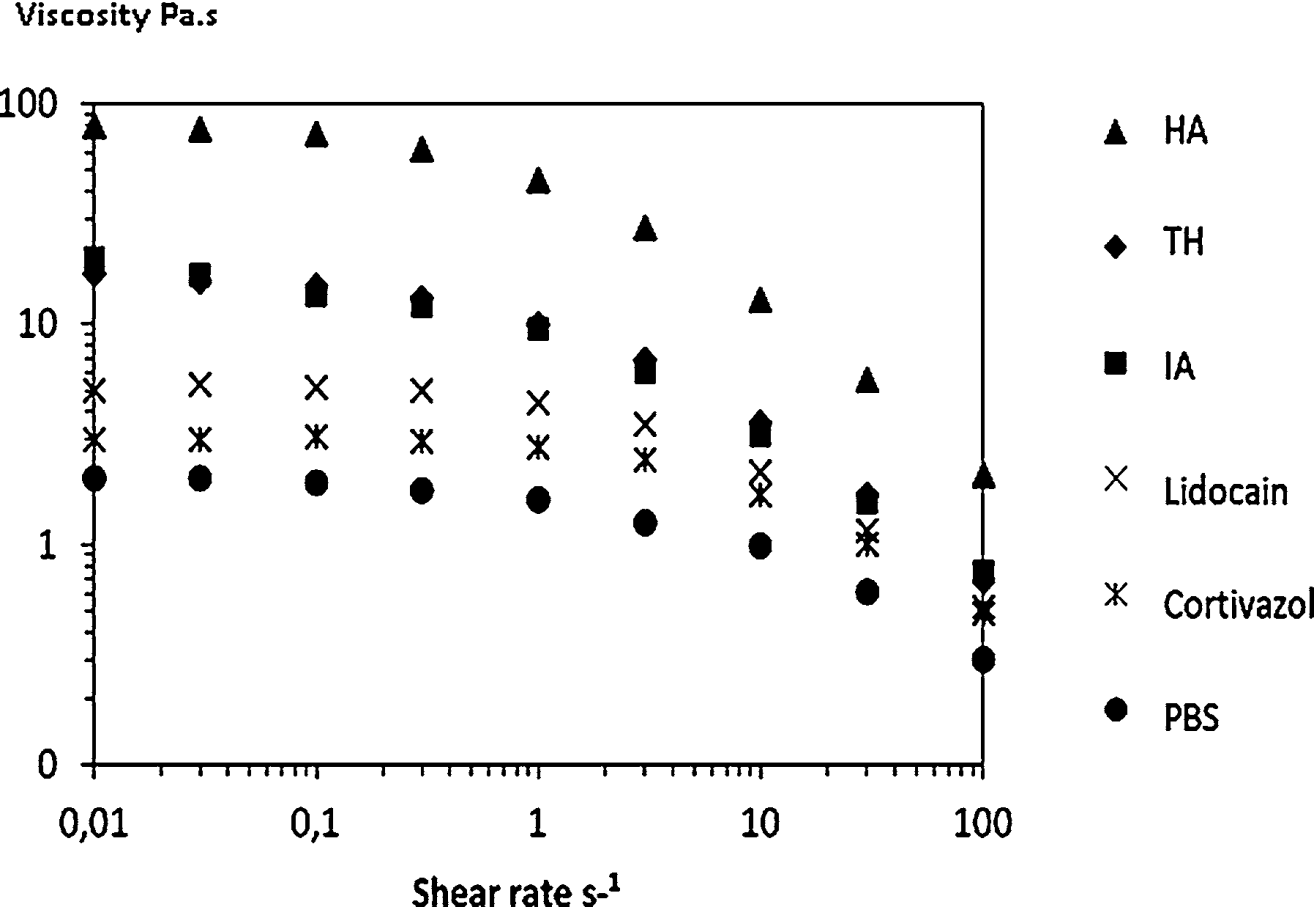
Flow curve of linear hyaluronic acid mixed with different products (ratio 1:1). *HA* hyaluronic acid, *TH* triamcinolone hexacetonide, *IA* ioxaglic acid, *PBS* phosphate buffer saline

Table 2 and Fig. 3 summarize the effect of the different molecules added to the linear viscosupplement. They point out the major effect of dilution on the HA rheological properties regardless of the added molecule. Cortivazol and lidocain have substantially the same effect as the stock dilution with PBS. However ioxaglic acid and even more triamcinolone hexacetonide reduced a much lower the viscosity of the HA solution, up to a 1:1 ratio.

**Table 2:** Influence of the diluent (in a ratio 1:1) on viscosity of HA solutions at 0.01 s^−1^ and 25 °C

HA structure	Viscosity at 0.01 s^−1^ (Pa.s)					
	HA	HA + PBS	HA + cortivazol	HA + TH	HA + lidocain	HA + IA
Linear HA	79	2	3	17	5	20
Cross-linked HA	2560	520	576	1450	306	359

*HA* hyaluronic acid, *TH* triamcinolone hexacetonide, *IA* ioxaglic acid, *PBS* phosphate buffer solution

**Fig. 4:**
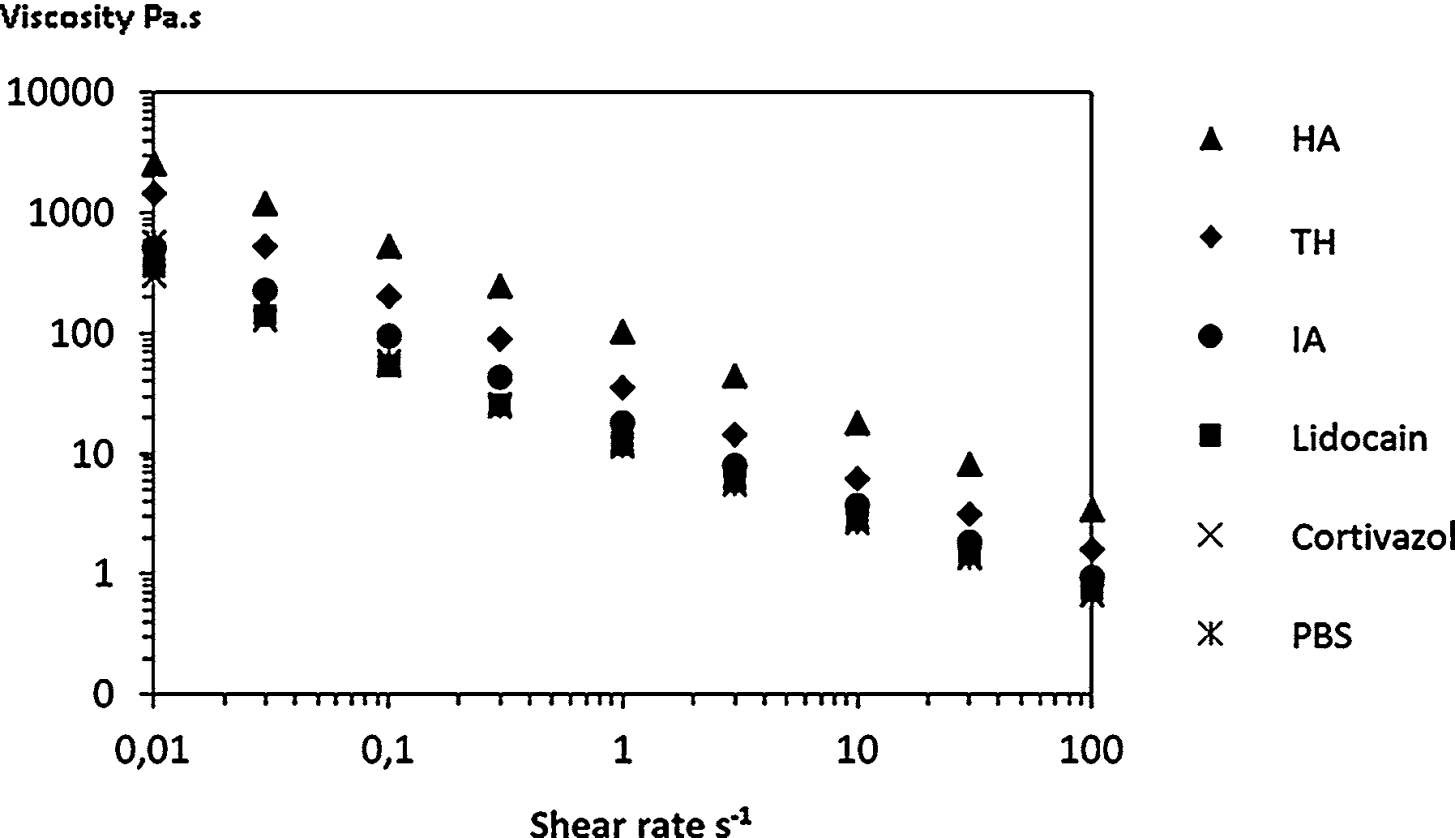
Flow curve of cross-linked hyaluronic acid mixed with different products (ratio 1:1). *HA* hyaluronic acid, *TH* triamcinolone hexacetonide, *IA* ioxaglic acid, *PBS* phosphate buffer saline

Table 3 and Fig. 4 summarize the effect of the different molecules added to the cross-linked HA. The dilution effect was much less pronounced because of cross-linking, holding the cohesion of the gel in aqueous media up to a certain limit. Here again, a significantly different effect of the two tested corticosteroids was noticed. Triamcinolone hexacetonide reduced moderately the viscosity unlike cortivazol, whereas the effect of the latter was roughly similar than that of PBS.

**Table 3:** Influence of dilution by phosphate buffer (PBS) and iodinated contrast agent, ioxaglic acid (IA), on hyaluronic acid (HA) viscosity (Pa.s at 0.01 s^−1^)

HA structure	Diluent	Viscosity at 0.01 s^−1^ (Pa.s)				
		HA	Ratio 1/0.5	Ratio 1/1	Ratio 1/2	Ratio 1/4
Linear HA	PBS	77	10	2	1	0.07
	IA	77	38	20	4	–*
Crosslinked HA	PBS	2560	1010	520	130	15*
	IA	2560	900	359	49	20*

* Phase separation

In contrast, lidocain hydrochloride and, even more ioxaglic acid, had a more marked effect on the cross-linked HA viscosity than on the linear one, most likely as a result of secondary interactions with HA due to the ionic charge and/or pH.

The effect of the iodinated contrast agent on the viscosity was clearly volume-dependent, and depended on the formulation of the viscosupplement. Up to a ratio of 1:1, the viscosity of the linear HA remained above the dilution effect. Beyond this ratio, ioxaglic acid had a similar effect to that of the buffer. On the cross-linked HA, the deleterious effect of the contrast agent appeared from 1:1 and became very significant at a 1:2 ratio. Visual observation shew that, regardless of the HA, there was a separation phase for the 1:4 ratio. At all dilution levels and regardless of the diluents, the viscosity of the cross-linked viscosupplement remained much higher than that of the linear HA.

## Discussion

The main lesson that can be drawn from this in vitro study is that addition of any other product to hyaluronic acid may greatly modify its rheological behaviour. Indeed, the rheological properties of HA are highly dependent on the product [concentration × molecular weight] of the latter (Fouissac et al. 1993). The effect of dilution is not negligible, in particular on the linear viscosupplement whose cohesion is greatly affected as soon as this dilution reaches a ratio of 1:0.5. The dilution of the drug is suspected to reduce its effectiveness. It is the reason why, it is usually advised to carefully remove any synovial effusion prior to an intra-articular injection (Maricar et al. 2013; Uthman et al. 2003). We had previously shown that adding a linear HA in an equivalent volume of an osteoarthritic synovial fluid was not sufficient to increase the viscosity of the latter. However a significant increase of the synovial fluid viscosity was observed when adding an equivalent volume and concentration of a cross-linked HA, suggesting the importance of interaction between exogenous HA and the three-dimensional network constituted by HA and synovial fluid proteins (Mathieu et al. 2009).

The second lesson is that the 2 tested corticosteroids, both frequently used in the treatment of OA, have differential effects on the viscosupplement properties. Cortivazol has no significant impact on HA properties. The decrease in viscosity is only slightly greater than that obtained with PBS. Conversely triamcinolone hexacetonide seems to stabilize the viscosupplement, suggesting that the increase in viscosity, results from the existence of favourable interactions between HA and triamcinolone. Thus, if a corticosteroid should be injected together with a viscosupplement, it seems logical to favour the second rather than the first, as long as it has not been demonstrated this does not affect the clinical outcome.

Similarly, lidocain, which cytotoxicity on chondrocytes is highlighted by numerous recent works (Rao et al. 2014; Ravnihar et al. 2014; Rahnama et al. 2013), does not seem to be used in combination with HA, despite the latter decreases its cytotoxicity (Onur et al. 2013). In our experiments lidocain has a deleterious effect on the cross-linked HA. Its effect on linear HA solution appears less clearly, probably because the simple dilution already causes a major loss of viscosity.

Finally, this study suggests that, in case of fluoroscopic guidance, the amount of iodinated contrast media must be as low as possible. In a ratio of 1:0.5, meglumine ioxaglate does not alter significantly the viscosity of the cross-linked HA, but as soon a ratio of 1: 1 (i.e. in clinical practice 2 ml of contrast for 2 ml of HA) the viscoelastic performances decrease dramatically, much beyond the dilution effect. For linear HA, viscosity of the mixture remains somewhat higher than that obtained with the PBS until a 1:2 ratio. Anyway it seems to us logical not to perform viscosupplementation during an arthrography for diagnosis purposes and to use only the minimum dose of contrast agent required to assert the intra-articular needle position (i.e. most of the time less than 1 ml). In a cohort of 50 patients with talo-crural OA (Conrozier et al. 2014), the success rate was 100 % among those injected under ultrasound control versus only 64.5 % among those injected using fluoroscopy guidance, leading to the hypothesis that this difference may result to the use of a contrast agent.

The main limitation of our study is due to the limited choice of the studied viscosupplements. Indeed, viscosupplements differ widely from each other so the results of clinical trials with a particular viscosupplement cannot be systematically extrapolated to others (Henrotin et al. 2015). It is possible that the results would be slightly different with viscosupplements of different molecular weight and or concentration, with other methods of cross-linking, or with viscosupplements not containing mannitol, which protects HA from reactive oxygen species-induced degradation (Rinaudo et al. 2014; Conrozier et al. 2014).

Prospective clinical trials are therefore needed to confirm or refute this hypothesis and to determine if the in vitro variations have an impact on the clinical outcome of viscosupplementation.
